# Feasibility of assessing vegetative and generative endpoints of crop- and non- crop terrestrial plant species for non-target terrestrial plant (NTTP) regulatory testing under greenhouse conditions

**DOI:** 10.1371/journal.pone.0230155

**Published:** 2020-03-10

**Authors:** Andreas Duffner, Thomas Moser, Marco P. Candolfi

**Affiliations:** 1 Depatment of Terrestrial Ecotoxicology, Eurofins Agroscience Services Ecotox GmbH, Niefern-Öschelbronn, Germany; 2 Eurofins Agroscience Services Ecotox GmbH, Niefern-Öschelbronn, Germany; Canakkale Onsekiz Mart University, TURKEY

## Abstract

Agriculture is the dominating land-use in the EU member states covering nearly half of the surface area. Using herbicides to reduce weed competition in agricultural areas can adversely affect Non-Target Terrestrial Plants (NTTP) growing in field margins. According to the EFSA Scientific Opinion on NTTPs an important protection goal is to maintain the biodiversity of plant species in agricultural areas. EFSA recommends to include also non-crop species mentioned in OECD guidelines (OECD 208 and 227) in the testing and to assess not only vegetative but also generative endpoints during the plant life-cycle such as flowering and seed production. The objectives of this study were to evaluate the feasibility of assessing generative endpoints of crop and non-crop species for NTTP regulatory testing under greenhouse conditions and to assess if generative endpoints are more sensitive than vegetative endpoints. The experimental design consisted of one control and four herbicide (Atlantis® WG) application rates, with 6 replicates each. The application rates of the test substance were the maximum field rate and 30%, 10% and 3% of the field rate. Biomass, plant height, flowering, seed production as well as seedling emergence of the F1 generation were assessed. The study shows a feasible approach to assess vegetative and generative endpoints of (non-) crops species under greenhouse conditions on the basis of the OECD guideline 227. The vegetative endpoints plant height and biomass were not more sensitive if assessed during the generative growth stage when compared to the vegetative growth stage of the plants. In contrast to that, the generative endpoint seed production was partly more sensitive in comparison to the vegetative endpoints biomass and plant height. For regulatory NTTP studies, 5 or more test substance rates at non-lethal levels should be tested so to allow the determination of ER_10/50_ values for vegetative and generative endpoints.

## Introduction

Terrestrial plants are providing a broad spectrum of ecosystem services such as the provision of food, natural medicines or the regulation of air quality [1]. In Europe, agriculture is the dominating land use covering nearly half of the surface area [2]. Plants in an agricultural ecosystem can be divided into three groups: crop plants, target plants for herbicides treatments (weeds) and non-target terrestrial plants (NTTP’s), these being non-crop plants in the off-crop area which should not be affected by any plant protection product (PPP) treatment [3, 4]. Using herbicides to reduce weed competition in cropped agricultural areas increases on the one hand crop productivity [5] and on the other hand may adversely affect NTTP’s, e.g. by reducing plant species richness, abundance and/or diversity in the adjacent habitats of crop fields such as field margins, hedgerows or ditches [6–8].

A risk assessment on PPPs side-effects and specially herbicide effects on NTTP´s is compulsory in the European Union (Regulation (EC) No 1107/2009) [9]. The aim is to reduce the ecological impact on NTTPs survival, seed production, plant diversity and so protect organisms such as insects, birds and bees which depend on these plants for their survival and development [10, 11]. For regulatory purposes, the potential side-effects of PPPs on NTTP´s is currently assessed under greenhouse conditions by assessing the effects treated soil on the NTTPs seedling emergence according to the OECD 208 guideline [3] and by assessing the effects on vegetative endpoints of sprayed PPPs on young potted crop plants according to the OECD 227 [4] guideline. Crop species are used as surrogates for wild off-crop plant species, since easier to cultivate. This is in line with the review article of Christl. et al. [12], which showed that there were no significant differences for the vegetative endpoints when comparing crops with non-crop species. In these studies, only vegetative endpoints such as plant height and biomass are measured. Recent studies however, indicate that generative endpoints such as seed production, may be more sensitive [11, 13, 14]. EFSA reported in their Scientific Opinion on NTTPs [15], that the protection goal is the maintenance of biodiversity of plant species in agricultural areas. EFSA, recommended to consider not only crop but also non-crop plant species in the testing and risk assessment scheme as well as to additionally assess generative endpoints such as flowering and seed production on top of vegetative endpoints (e.g. biomass).

The ISO guideline 22030 [16] was developed as seedling emergence test to assess vegetative (biomass) and generative endpoints (flowers and/or seed production) of the two crop species, turnip rape (*Brassica rapa*) and oat (*Avena sativa*). Tarazona *et al*. [17] compared the OECD 208 with the ISO 22030 guideline using probabilistic models with the aim to quantify the sensitivity of the test systems. The modelling results suggested that an OECD 208 test with six NTTP species compensates for the higher sensitivity of the generative endpoints assessed with the ISO 22030 test. The ISO protocol is however, limited to the exposure of only two crop species at the seeding stage.

Currently, there are only few published studies where standardized and validated protocols where used to study the effects of PPPs on vegetative as well as generative endpoints of non-crop species under greenhouse conditions [18]. Brain and Hoberg [18] exposed ten terrestrial plants, to a direct overspray of atrazine according to seedling emergence and vegetative vigor study guidelines and evaluated the potential for recovery. They found that in most species where initial herbicidal effects were observed, the effects are largely ameliorated over time.

The objective of this work was to assess a) if standardized and validated test protocols designed for crop species can be adapted/used also for non-crop plant species testing, b) the feasibility of assessing generative endpoints of crop and non-crop NTTP species for regulatory testing under greenhouse conditions with regard to labor, duration of the experiment and success rate and c) if vegetative endpoints (plant height and biomass) differ if assessed during the vegetative or generative phase of the study.

## Material and methods

The study was conducted in a greenhouse located in Neulingen-Göbrichen, Germany. Eighteen plant species, 15 dicotyledonous and 3 monocotyledonous, representing 11 different plant families were tested. Ten out of the 18 plant species were non-crop species (Table 1).

**Table 1:** Crop and non-crop plant species selected for the conduct of the study.

Species name	Common name	Family
**Non-Crop Species**		
*Agrostemma githago*	Corn-cockle	*Caryophyllaceae*
*Avena fatua*	Wild oat	*Poaceae*
*Chenopodium berlandieri*	Pitseed goosefoot	*Amaranthaceae*
*Coriandrum sativum*	Chinese parsley	*Apiaceae*
*Leucanthemum vulgare*	Oxeye daisy	*Asteraceae*
*Lotus corniculatus*	Bird's-foot trefoil	*Fabaceae*
*Matricaria recutita*	Chamomile	*Asteraceae*
*Papaver rhoeas*	Common poppy	*Papaveraceae*
*Phacelia tanacetifolia*	Lacy phacelia	*Boraginaceae*
*Trifolium pratense*	Red clover	*Fabaceae*
*Veronica persica*	Birdeye speedwell	*Plantaginaceae*
*Vicia sativa*	Vetch	*Fabaceae*
**Crop Species**		
*Brassica rapa*	Turnip	*Brassicaceae*
*Lepidium sativum*	Garden cress	*Brassicaceae*
*Fagopyrum esculentum*	Buckwheat	*Polygonaceae*
*Lolium multiflorum*	Italian rye-grass	*Poaceae*
*Secale cereale*	Rye	*Poaceae*
*Sinapis alba*	White mustard	*Brassicaceae*

To test the sensitivity of the plants a control (tap water) and 4 different treatment levels of an herbicide were used. Each treatment consisted of 6 replicates (pots). Four and eight seeds per pot were used for the dicotyledonous and monocotyledonous species, respectively, due to the different biomass production between dicotyledonous and monocotyledonous species. On December 28, 2018 untreated seeds purchased from 6 commercial suppliers in Germany (Bingenheimer Saatgut (Echzell), Templiner Kräutergarten (Templin), Wildsameninsel (Temmen-Ringenwalde), WeberSeeds (Vaals) and Hild Samen (Marbach am Neckar)) were planted at a depth of 0.5 to 1 cm and equally spaced in each pot (diameter: 15 cm, height: 11 cm) filled with approximately 1.3 kg soil. The soil (Supplier: EBRD GmbH & Co. KG, Germany) was a loamy sand with a pH of 7.5 (measured in 0.01 M CaCl_2_) and 0.23% organic carbon. The greenhouse is equipped with LED lamps (FL300; Senmatic). Light was automatically regulated to guarantee a photo period of 16 hours with a minimum light intensity of 300 μmol m^-2^ s^-1^. Air temperature and relative air humidity in the greenhouse were measured continuously with an integrated monitoring system in the shade at plant height. Regular irrigation with tap water was provided. Fertilisation with a 0.2% nutrient NPK solution (Hakaphos® Blau; Compo Expert; Münster) was performed weekly.

As test substance, the herbicide Atlantis® WG (Bayer CropScience, two active ingredients: 30 g mesosulfuron-methyl kg^-1^; 6 g iodosulfuron-methyl-natrium kg^-1^ and asafener: 90 g mefenpyr-diethyl kg^-1^, mode of action: inhibition of plant cell division) was used. This herbicide can be used to control grass and annual broad leaved weeds in winter, spring and durum wheat, triticale and rye grass [19]. The herbicide application is recommended to be performed once the crop reached the growth stage of 2–4 true leaves (BBCH 12–14 [20]).

The treatments with the respective application rates and their proportion of the recommended field rate is given in Table 2. Treatments were the same as in the field study performed by Mack *et al*. [21].

**Table 2:** Treatments with the test substance Atlantis^®^ WG.

Treatment	Application rate of Atlantis^®^ WG g ha^-1^	Proportion of recommended field rate %
C (Control)	-	-
T1	12	3
T2	40	10
T3	120	30
T4	400	100

Tap water was used as the solvent for the test substance. The highest test substance application solution served as a stock solution. For all lower application rates aliquots were taken and diluted in tap water. All applications were carried out at a spray volume of 200 L water ha^-1^. The application was conducted with a laboratory track-sprayer (Schachtner, Ludwigsburg, Germany) using a 80015 EVS nozzles (TeeJet, Ludwigsburg). The distance between nozzle and the plants tip was 43 cm.

Samples of the application solutions were stored deep frozen (-18°C) until analytical dose verification. The analytical dose verification for both active ingredients (mesosulfuron-methyl and iodosulfuron-methyl-natrium) was performed by HPLC/MS/MS for the highest test substance treatment level (T4) and the control test solutions.

**Fig 1:**
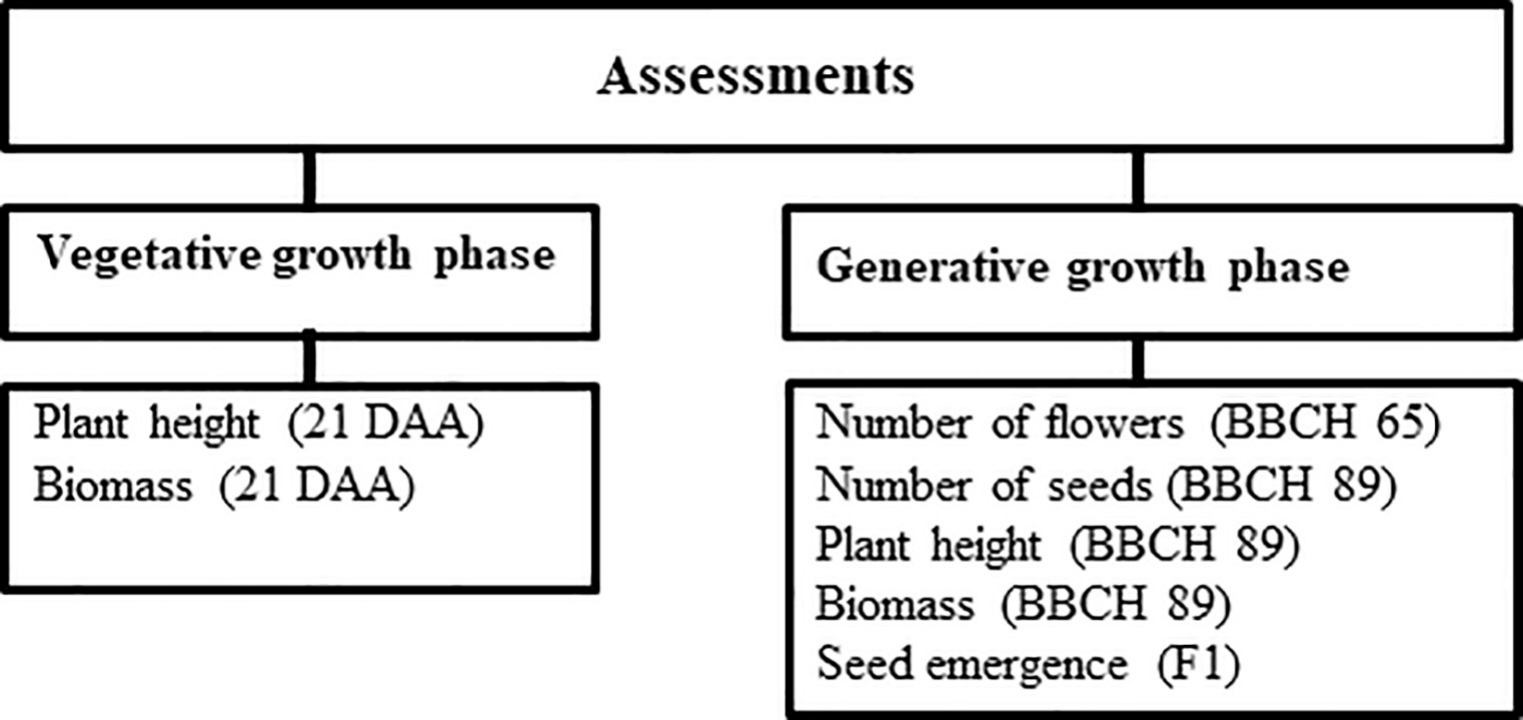
Assessments performed at the vegetative and generative growth stage of the plants (DAA = Days After Application).

An overview of the vegetative and generative endpoints assessments performed during the study is presented in Fig 1. Plant height of the surviving plants (from the soil surface to the apical tip or highest aerial part of the plant) was measured 21 days after application (DAA) and at BBCH 89 (generative growth stage, fully ripe plants). The biomass (dry weight) of the plants was determined by cutting the surviving plants at soil level at 21 DAA during the vegetative growing phase of the plants and when the plants reached the fully ripe stage (BBCH 89) during generative growth phase. At the first assessment, half of the plants per replicate were cut; the remaining plants were assessed at growth stage BBCH 89. The plants were dried at 60°C in a laboratory-type drying cabinet for 48 hours. Average dry weight per plant was calculated by dividing the dry weight by the number of surviving plants of the replicate. Mean values and standard deviations were determined for each treatment.

Number of flowers was assessed for all treatments when the control reached BBCH 65 (full flowering). Number of seeds of each species and treatment level was counted separately when reaching the appropriate growth stage (BBCH 89). Seed production was not assessed for treatments where seed development was not completed 4 weeks after the control reached BBCH 89. Before evaluating the germination rate of the F1, the harvested seeds were stored in a paper bag in the fridge (5.1–6.9°C) for 6 months. To assess germination rate, 3 replicates per treatment and species were used. Ten seeds per replicate were cultivated in a similar soil and pots as previously described. Plant species from which insufficient number of seeds could be collected were not included in the germination test.

Data on the plant biomass, plant height, number of flowers and seeds as well as emergence rate of the harvested seeds were evaluated statistically using ToxRat® [22]. The data were tested for normality and homoscedasticity using Shapiro-Wilk`s Test and Levene-Test followed by William`s test in case the data were normally distributed and homoscedasticity was given. Multiple Welch`s t-test with Bonferroni-Holm adjustment was conducted in case that the data were normally distributed but non-homogenous. In case the data were non-homogenous and not normally distributed, the Jonckheere-Terpstra test was used [23] to compare treatments effects. The significance level was set to α = 0.05 for all tests (one sided smaller). ER_50_ and their 95% confidence limits were determined by Probit analysis using linear max. likelihood regression, where possible [23].

## Results

### Environmental test conditions and analytical dose verification

The environmental conditions recorded during the course of the experiment were 20 and 32°C for temperature and 50 to 85% for relative humidity.

Analytical dose verification indicated a recovery of the two active ingredients between 92 and 106% in the highest treatment level (T4) and 0% in the control.

### Plant height and biomass (vegetative endpoints)

The plant height and the biomass assessed at 21 DAA and BBCH 89 could be evaluated for 11 out of the 18 species. Data of the remaining 7 plant species are not presented because at the generative growth stage plant height and biomass could not be assessed since also in the control treatment the species did not reach the generative phase within 4 months or the species did not reach BBCH 89 due to lack of seed formation (Table 3).

**Table 3:** Duration (days) of the different growth stages for each plant species.

Species	From seeding to BBCH 12–14 (test substance application)	BBCH 12–14 to BBCH 65 (flowering) of control treatment	BBCH 12–14 to BBCH 89 (fully ripe) of control treatment	From seeding to BBCH 89 of control treatment	Comments
	Duration in days				
**Non-crop species**					
*Agrostemma githago*	14	53	96	110	-
*Avena fatua*	26	34	49	75	-
*Chenopodium berlandieri*	14	-	-	-	No formation of seeds; Probably missing pollination
*Coriandrum sativum*	26	62	94	120	-
*Leucanthemum vulgare*	14	-	-	-	No generative phase after 4 months
*Lotus corniculatus*	26	49	-	-	No formation of seeds; Probably missing pollination
*Matricaria recutita*	20	33	90	110	Counting of seeds not feasible
*Papaver rhoeas*	14	49	96	110	Counting of seeds not feasible
*Phacelia tanacetifolia*	20	23	78	98	-
*Trifolium pratense*	20	43	92	112	No formation of seeds; Probably missing pollination
*Veronica persica*	20	-	-	-	No generative phase after 4 months
*Vicia sativa*	14	25	60	74	-
**Crop Species**					
*Brassica rapa*	20	-	-	-	No generative phase after 4 months
*Lepidium sativum*	14	39	67	81	-
*Fagopyrum esculentum*	26	27	52	78	-
*Lolium multiflorum*	20	-	-	-	No formation of seeds; Probably missing pollination
*Secale cereale*	20	-	-	-	No generative phase after 4 months
*Sinapis alba*	20	21	86	106	-

**Fig 2:**
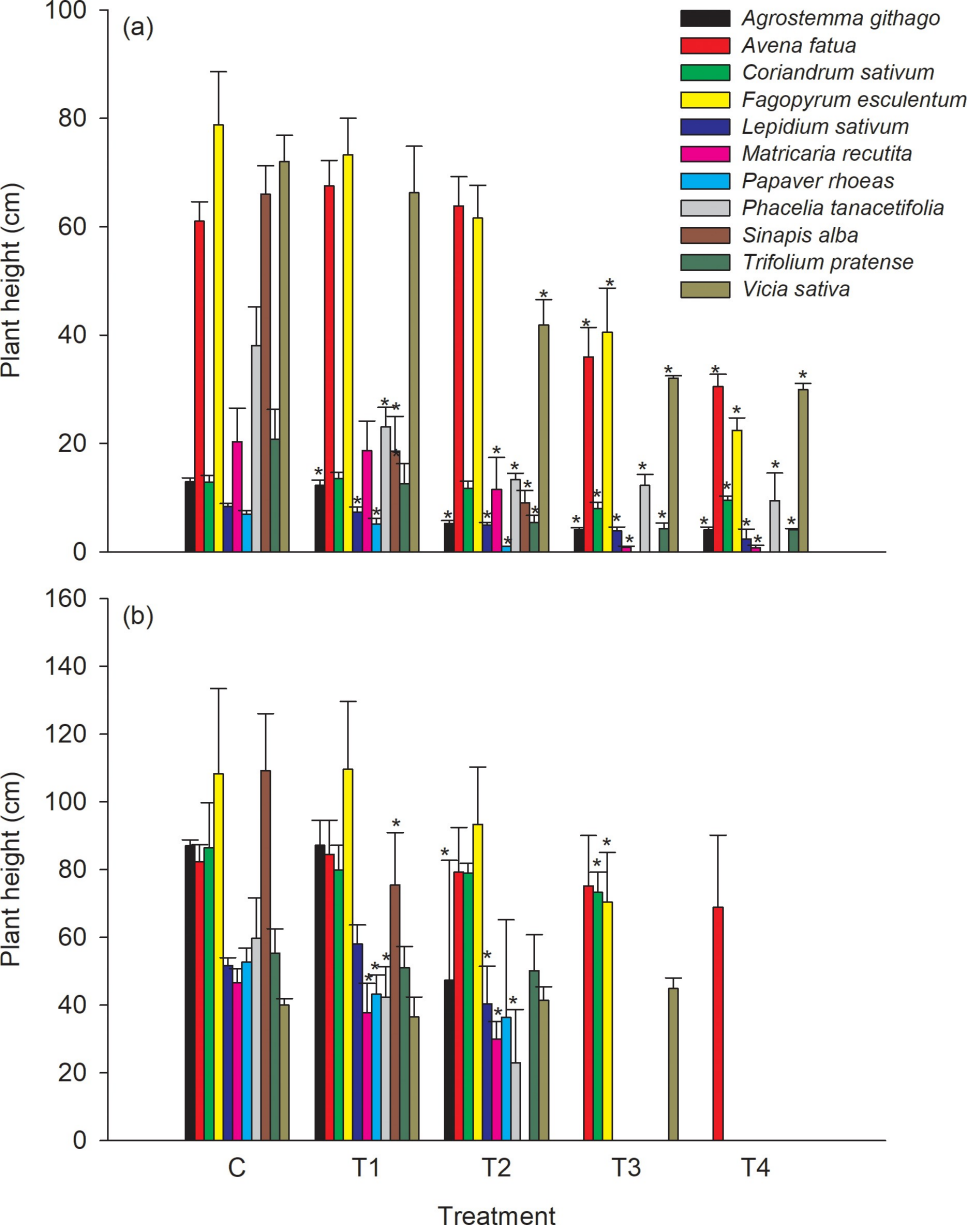
Mean plant height at the vegetative (21 DAA, (a)) and generative (BBCH 89, (b)) growth stage of the test plants, respectively. Error bars indicate the standard deviation and * indicate a statistical significant difference to the control (William`s test or Multiple Welch`s t-test with Bonferroni-Holm adjustment depending on homoscedasticity) for each plant species (α = 0.05, one sided). Missing columns within a treatment group indicates that no data could be assessed for the respective plant species.

**Fig 3:**
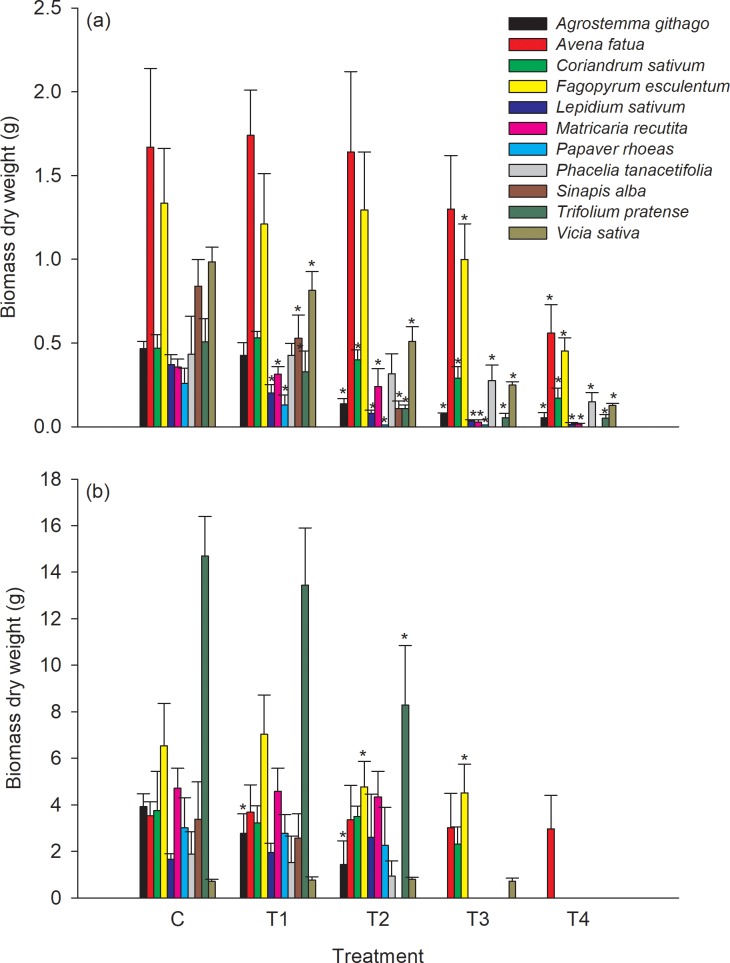
Mean biomass (dry weight) per plant at the vegetative (21 DAA, (a)) and generative (BBCH 89, (b)) growth stage, respectively. Error bars indicate the standard deviation and * indicate a statistical significantly difference to the control (William`s test or Multiple Welch`s t-test with Bonferroni-Holm adjustment depending on homoscedasticity) for each plant species (α = 0.05, one sided smaller). Missing columns within a treatment group indicates that no data could be assessed for the respective plant species.

At 21 DAA, effects on plant height and biomass were observed at the 2 highest treatment application rates T3 (30% of the field rate) and T4 (max field application rate) (Fig 2A and Fig 3A). Plant height of all species was statistically significantly lower when compared to the control at the two highest test substance application rates. *Agrostemma githago*, *Lepidium sativum*, *Papaver rhoeas*, *Phacelia tanacetifolia*, *Sinapis alba* and *Trifolium pratense* showed statistically significantly lower growth when compared to the control also down to the lowest test substance application rate of 3% of the field rate.

The results for biomass showed similar patterns. All species showed statistically significantly lower biomass when compared to the control at the two highest application rates of the test substance except for *Avena fatua* (Fig 2A and Fig 3A).

At the growth stage BBCH 89 the negative effects on plant height and biomass were smaller compared to the effects recorded during the vegetative growth phase at 21DAA (Fig 2 and Fig 3).

The ER_50_ for plant height could only be calculated for 6 species and the ER_50_ for plant biomass for 7 species, due to the very low or no plant survival at the two highest treatment application rates (T3 and T4) of the test substance (Table 4). For plant height, *Agrostemma githago* and *Lepidium sativum* showed a higher ER_50_ at growth stage 21 DAA than at BBCH 89. *Fagopyrum esculentum*, *Matricaria recutita* and *Papaver rhoeas* showed a lower ER_50_ at growth stage 21 DAA than at BBCH 89. For *Phacelia tanacetifolia* the ER_50_ was similar at both growth stages (Table 4). For biomass, *Phacelia tanacetifolia* showed a higher ER_50_ at growth stage 21 DAA than at BBCH 89. *Fagopyrum esculentum*, *Lepidium sativum*, *Papaver rhoeas* and *Trifolium pratense* showed a lower ER_50_ at growth stage 21 DAA than at BBCH 89. For *Agrostemma githago* and *Coriandrum sativum* the ER_50_ was similar at both growth stages.

**Table 4:** Effect of Atlantis WG on plant height and biomass (expressed as ER_50_ (with 95% confidence limits) recorded at 21 DAA (vegetative growth phase) and fully ripe stage of the plants BBCH 89 (generative growth phase) of the study.

Species	Plant height		Biomass (dry weight)	
	21 DAA	BBCH 89	21 DAA	BBCH 89
	ER_50_ (95% confidence limits) in g product ha^-1^			
**Non-crop species**				
*Agrostemma githago*	59 (38–90)	43 (n.d.) ^4^	29 (23–36)	25 (17–39)
*Avena fatua*	318 (242–467)	n.d. ^1)^	253 (188–366)	n.d. ^1)^
*Coriandrum sativum*	n.d. ^1)^	n.d. ^1)^	212 (165–286)	270 (133 –n.d. ^4^)
*Matricaria recutita*	41 (30–55)	94 (46 –n.d. ^4^)	49 (39–61)	n.d. ^1)^
*Papaver rhoeas*	19 (16–23)	146 (n.d.) ^4^	12 (2–19)	118 (n.d.) ^4^
*Phacelia tanacetifolia*	20 (10–32)	26 (18–64)	189 (114–438)	36 (n.d.) ^4^
*Trifolium pratense*	16 (7–25)	n.d. ^1)^	17 (12–23)	48 (33–254)
*Vicia sativa*	129 (92–193)	n.d. ^1)^	46 (39–54)	n.d. ^1)^
**Crop species**				
*Fagopyrum esculentum*	140 (118–169)	268 (136 –n.d.)	249 (176–400)	411 (n.d.)
*Lepidium sativum*	95 (64–145)	63 (n.d.) ^4^	14 (11–16)	115 (n.d.) ^4^
*Sinapis alba*	n.d. ^2)^	n.d. ^3)^	16 (12–20)	n.d. ^3)^

n.d. = not determined

^1)^ Effects were < 50%

^2)^ ER_50_ calculation not possible, because the effects of the assessed treatments (T1 and T2) are already > 70%

^3)^ ER_50_ calculation not possible, because only C and T1 could be assessed

^4)^ Confidence interval could not be calculated reliably

The NOER values (Table 5) for the vegetative endpoints plant height and biomass at the vegetative growth stage (21DAA) and generative growth stage (BBCH 89) showed a similar pattern regarding sensitivity as the ER_50_ values.

**Table 5:** NOER of plant species where height and biomass could be assessed during the vegetative (21 DAA) and generative (BBCH 89) growth stage, respectively.

Species	Plant height		Biomass (dry weight)	
	21 DAA	BBCH 89	21 DAA	BBCH 89
	NOER (in g product ha^-1^)			
**Non-crop species**				
*Agrostemma githago*	< 12	12	12	< 12
*Avena fatua*	40	≥ 400	120	≥ 400
*Coriandrum sativum*	40	40	12	≥ 120
*Matricaria recutita*	12	< 12	< 12	≥ 40
*Papaver rhoeas*	< 12	< 12	< 12	≥ 40
*Phacelia tanacetifolia*	< 12	< 12	40	≥ 40
*Trifolium pratense*	< 12	≥ 40	< 12	12
*Vicia sativa*	12	≥ 120	< 12	≥ 120
**Crop species**				
*Fagopyrum esculentum*	12	40	40	12
*Lepidium sativum*	< 12	12	< 12	≥ 40
*Sinapis alba*	< 12	< 12	< 12	< 12

### Number of flowers and seeds (generative endpoints)

**Fig 4:**
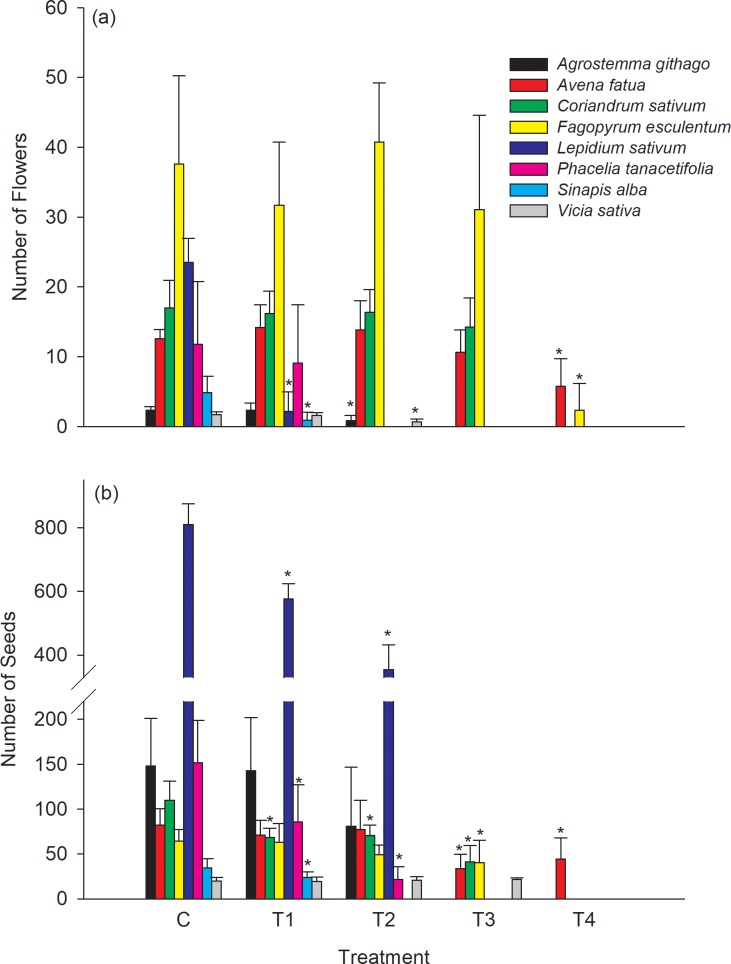
Mean number of flowers per plant at BBCH 65 (a) and seeds at BBCH 89 (b), respectively. Error bars indicate the standard deviation and * indicate a statistical significantly difference to the control (William`s test or Multiple Welch`s t-test with Bonferroni-Holm adjustment depending on homoscedasticity) for each plant species (α = 0.05, one sided smaller). Missing columns within a treatment group indicates that no data could be assessed for the respective plant species.

The number of flowers and seeds could be evaluated for 8 out of the 18 species (Fig 4). The reasons for not evaluating the remaining species were the lack of seed formation probably due to missing or insufficient pollination, counting of seeds was not feasible due to the size and/or number or the end of the generative phase was not reached after 4 months of study duration after the test substance application. The detailed reason are described in Table 3.

The number of flowers showed weaker effects at higher test application rates than the number of seeds (Fig 4). At the lowest treatment application rate of 3% of the field rate (T1), the number of flowers of *Lepidium sativum and Sinapis alba* and the number of seeds of *Coriandrum sativum*, *Phacelia tanacetifolia* and *Sinapis alba* were statistically significantly lower when compared to the control. At the highest treatment application rate (T4) only *Avena fatua* and *Fagopyrum esculentum* could be assessed for the number of flowers and only *Avena fatua* for the number of seeds. The assessed differences in these species, indicated statistically significantly lower generative endpoints in T4 when compared to the control.

For the 4 plant species *Chenopodium berlandieri*, *Lolium multiflorum*, *Lotus corniculatus* and *Veronica persica* no formation of seeds was observed, probably due to missing pollination.

Due to the strong effects at the two highest application rates of the test substance on plant survival the ER_50_ for flower and seed formation could not be calculated.

### Germination of harvested seeds (F1 generation)

The germination of the F1 generation could be assessed for the 7 plant species, for which sufficient seeds could be harvested, namly *Agrostemma githago*, *Avena fatua*, *Coriandrum sativum*, *Fagopyrum esculentum*, *Lepidium sativum*, *Phacelia tanacetifolia*, *Sinapis alba and Vicia sativa* (Table 6). At the second and third highest application rate (T2 and T3), the 2 non-crop species *Phacelia tanacetifolia* and *Vicia sativa* had a statistically significantly lower germination rate when compared to the control. In contrast, *Avena fatua* and *Coriandrum sativum* showed a higher germination rate at all test substance treatments when compared to the control. The other 4 species did not show any differences in the germination rate between the test substance treatments and the control (Table 6).

**Table 6:** Germination rate in % of harvested seeds (F1).

Species	Emergence (%)				
	Application rates of test substance (g product ha^-1^)				
	0 (Control)	12	40	120	400
**Non-crop species**					
*Agrostemma githago*	100	97	97	n.d.	n.d.
*Avena fatua*	20	30	53	70	67
*Coriandrum sativum*	27	63	57	67	n.d.
*Phacelia tanacetifolia*	87	77	67 *	n.d.	n.d.
*Vicia sativa*	90	90	93	57 *	n.d.
**Crop species**					
*Fagopyrum esculentum*	97	100	100	100	n.d.
*Lepidium sativum*	100	100	100	n.d.	n.d.
*Sinapis alba*	100	100	n.d.	n.d.	n.d.

^*^ statistical significantly difference to the control for each plant species (α = 0.05, one sided)

n.d. = value could not be determined.

### Study duration

Study duration from sowing until fully ripening of the plant seeds (BBCH 89) of the control plant species was in average 98 days ranging from 75 to 120 days (Table 3).

## Discussion

This study showed that the assessment of vegetative as well as generative endpoints of crop and especially non-crop plant species for regulatory testing under greenhouse conditions is in general feasible with regard to labor, duration of the experiment (Table 3) and success rate (e.g. germination rate of non-crop species (S2 Text)), which were the main objectives of this study.

Eleven out of 18 plant species (including 6 non-crop species) could be used to compare vegetative endpoints during the vegetative and generative growth phase of the plants, and 8 out of 18 (including 5 non-crop plant species) could be used to assess flowering and seed production. The germination rate of the evaluated species was ≥ 70% (S2 Text), which fulfils the validity criteria of the OECD guideline 227 [4]. Control plant species reached fully ripening of the seeds (BBCH 89) in average after 98 days (ranging from 75 to 120 days), which is a practicable and not too long test duration. For the 4 species *Chenopodium berlandieri*, *Lolium multiflorum*, *Lotus corniculatus* and *Veronica persica* no formation of seeds could be observed, probably due to missing pollination. For the 2 species *Matricaria recutita* and *Papaver rhoeas* counting the seeds was challenging due the high number and the small size of the seeds. Including seed weight as additional parameter in upcoming studies could enable the assessment of seed production especially of plant species with numerous and small seeds.

Sensitivity ranking of the tested species is similar if the NOER (Table 5) or the ER_50_ values (Table 4) of the vegetative endpoints plant height and biomass recorded 21 days after test substance application (vegetative growth stage), and at BBCH 89 (generative growth stage), are evaluated. In the cases where the plants survived until the assessment of the generative endpoints, the biomass of all species except of *Agrostemma githago*, *Fagopyrum esculentum* and *Phacelia tanacetifolia* and the plant height of all species except of *Coriandrum sativum*, *Matricaria recutita*, *Papaver rhoeas*, *Phacelia tanacetifolia* and *Sinapis alba* had a higher NOER at BBCH 89 than 21DAA, respectively (Table 5, Fig 2, Fig 3). This decrease of the measured effects indicates a recovery effect of the plants of the vegetative endpoints, plant height and biomass.

Similar results were observed in greenhouse by Brain and Hoberg [18], and Carpenter and Boutin [24] and Nelemans *et al*. [25] under field conditions. In the greenhouse, Brain and Hoberg [18] recorded a clear recovery in biomass in 7 of 9 crop species after treatment with atrazine (at 2- to 4-leaf stage) between days 0–21 and 21–42 days after treatment application. Carpenter and Boutin [24], observed also a recovery in biomass over time for wild plants after treatment with glufosinate ammonium.

However, our results show a dose response to the treatment with Atlantis® WG, where irreversible effects increased with increasing application rates. The increase in effects over time is explained by the mode of action of the active ingredients inhibiting biosynthesis of essential amino acids. Due to that reason the assessments at the generative growth stage have been not possible for some species, in particular at the higher application rates.

For plant species were biomass as well as seed production could be evaluated, 3 species (*Avena fatua*, *Coriandrum sativum* and *Phacelia tanacetifolia*) had a lower, 3 species (*Fagopyrum esculentum*, *Lepidium sativum* and *Sinapis alba*) had a similar and 2 species (*Agrostemma githag*, *Vicia sativa*) had a higher NOER for seed production compared to biomass (Table 5, Fig 3, Fig 4B). Similar results were observed in other greenhouse studies. Boutin *et al*. [11] assessed generative endpoints (e.g. seed production) mainly of non-crop species and observed that overall, the generative endpoints were more sensitive in 58% of the plant species (34 out of 59 species) whereas vegetative endpoints were more sensitive in 32% of the plants species. Andersson [26] observed similar effects for three non-crop species in a greenhouse study.

Flowering, expressed as number of flowers, was for most species less sensitive and more variable when compared to seed production. For *Agrostemma githago*, *Lepidium sativum*, *Sinapis alba* and *Vicia sativa* significant differences were detected at the two lowest rates when compared to the control. A similar response was also observed in field studies [11, 27].

The germination of harvested seeds (F1) was assessed as an indicator of potential shifts in species composition and succession of the vegetation [27] and of higher frequencies of more tolerant species [28]. No clear trend was found regarding the influence of the treatment rates of Atlantis® WG on the germination rates (Table 6). The germination rate of the control groups of each species, except of *Avena fatua* and *Coriandrum sativum*, was ≥ 87%. Especially for non-crop species testing of the F1 generation, dormancy and required pretreatments (e.g. stratification) of seeds needs to be considered to achieve an optimal germination under greenhouse conditions [27, 29]. The germination results presented in Table 6 were achieved after a storage period of 6 months in the fridge. A subset of the harvested seeds was sown within 14 days after the harvest which resulted in low germination rates also in the control for most species (S2 Text). This indicates that the longer storage and preparation of harvested seeds is crucial to obtain reliable study results.

Since the highest tested application rate (field rate) caused 100% mortality in most of the species tested (S1 Text), the calculation of an ER_x_ value for the generative endpoints was not possible. Further studies with the objective to determine generative endpoints for regulatory testing of non-target terrestrial plants should aim to determine ER_10/50_ values. The ER_10/50_ values are more suitable to compare the sensitivity of vegetative and generative endpoints and so to be able to determine the most sensitive endpoint [30]. It is therefore, essential to test non-lethal application rates of the test substance eventually even by performing pre-tests to determine the appropriate testing rate for each species. The repeatability of this study design will be evaluated after conducting further studies with this study objective. The statistical evaluation can then be extended and validated. For future research a standardized description of the trait characteristics especially of non-crop species is suggested. This would allow to extrapolate or to compare the observations with other plant species or studies.

## Conclusion

Vegetative and generative endpoints of crop and non-crops species can be assessed under greenhouse conditions on the basis of the OECD guideline 227. The vegetative endpoints plant height and biomass were not more sensitive if assessed during the generative growth stage when compared to the vegetative growth stage of the plants. In contrast to that, the generative endpoint seed production was partly more sensitive in comparison to the vegetative endpoints biomass and plant height. For regulatory NTTP studies, five or more test substance rates at non-lethal levels should be tested so to allow the determination of ER_10/50_ values for vegetative and generative endpoints.

## Supporting information

S1 Text Mortality data. (DOCX)

S2 Text Emergence data. (DOCX)
