# Supplementary material for: Feasibility of assessing vegetative and generative endpoints of crop- and non- crop terrestrial plant species for non-target terrestrial plant (NTTP) regulatory testing under greenhouse conditions
Source: PLoS One. 2020 Mar 10;15(3):e0230155. doi: 10.1371/journal.pone.0230155 (PMC7064193; doi:10.1371/journal.pone.0230155)
Supplement: S1 Text — (DOCX) [file pone.0230155.s001.docx]

Table B. Mortality of the different plant species at the vegetative growth stage (21 DAA). Mortality is given %.

| **Species** | **Application rates of test substance** | | | | | |
| --- | --- | --- | --- | --- | --- | --- |
|  | **(g product ha^-1^)** | | | | | |
|  | **0 (Control)** | | **12** | **40** | **120** | **400** |
| *Avena fatua* | 0.0 | 0.0 | | 0.0 | 0.0 | 0.0 |
| *Brassica rapa* | 0.0 | 0.0 | | 0.0 | 12.5 | 45.8 |
| *Chenopodium berlandieri* | 0.0 | 0.0 | | 0.0 | 8.3 | 100.0 |
| *Coriandrum sativum* | 0.0 | 0.0 | | 0.0 | 0.0 | 4.2 |
| *Fagopyrum esculentum* | 0.0 | 0.0 | | 0.0 | 0.0 | 0.0 |
| *Lepidium sativa* | 0.0 | 0.0 | | 0.0 | 37.5 | 54.2 |
| *Leucanthemum vulgare* | 0.0 | 0.0 | | 16.7 | 29.2 | 50.0 |
| *Lolium multiflorum* | 0.0 | 0.0 | | 0.0 | 0.0 | 37.5 |
| *Lotus corniculatus* | 0.0 | 0.0 | | 0.0 | 0.0 | 29.2 |
| *Matricaria recutita* | 0.0 | 0.0 | | 0.0 | 8.3 | 25.0 |
| *Papaver rhoeas* | 0.0 | 0.0 | | 8.3 | 75.0 | 100.0 |
| *Phacelia tanacetifolia* | 0.0 | 0.0 | | 4.2 | 0.0 | 29.2 |
| *Secale cereale* | 0.0 | 0.0 | | 0.0 | 0.0 | 0.0 |
| *Sinapis alba* | 0.0 | 0.0 | | 33.3 | 100.0 | 100.0 |
| *Trifolium pratense* | 0.0 | 0.0 | | 0.0 | 33.3 | 20.8 |
| *Veronica persica* | 0.0 | 0.0 | | 0.0 | 4.2 | 8.3 |
| *Vicia sativa* | 0.0 | 0.0 | | 0.0 | 0.0 | 0.0 |

Table C. Mortality of the different plant species at the generative growth stage (BBCH 89). Mortality is given %.

| **Species** | **Application rates of test substance** | | | | |
| --- | --- | --- | --- | --- | --- |
|  | **(g product ha^-1^)** | | | | |
|  | **0 (Control)** | **12** | **40** | **120** | **400** |
| *Avena fatua* | 0.0 | 0.0 | 0.0 | 0.0 | 0.0 |
| *Brassica rapa* | n.d. | n.d. | n.d. | n.d. | n.d. |
| *Chenopodium berlandieri* | n.d. | n.d. | n.d. | n.d. | n.d. |
| *Coriandrum sativum* | 0.0 | 0.0 | 0.0 | 0.0 | 8.3 |
| *Fagopyrum esculentum* | 0.0 | 0.0 | 0.0 | 0.0 | 0.0 |
| *Lepidium sativa* | 0.0 | 0.0 | 0.0 | 41,7 | 66.7 |
| *Leucanthemum vulgare* | n.d. | n.d. | n.d. | n.d. | n.d. |
| *Lolium multiflorum* | n.d. | n.d. | n.d. | n.d. | n.d. |
| *Lotus corniculatus* | n.d. | n.d. | n.d. | n.d. | n.d. |
| *Matricaria recutita* | 0.0 | 0.0 | 0.0 | 16.7 | 41.7 |
| *Papaver rhoeas* | 0.0 | 0.0 | 16.7 | 50.0 | -* |
| *Phacelia tanacetifolia* | 0.0 | 0.0 | 8.3 | 0.0 | 50.0 |
| *Secale cereale* | n.d. | n.d. | n.d. | n.d. | n.d. |
| *Sinapis alba* | 0.0 | 0.0 | 50.0 | -* | -* |
| *Trifolium pratense* | 0.0 | 0.0 | 0.0 | 33.3 | 25.0 |
| *Veronica persica* | n.d. | n.d. | n.d. | n.d. | n.d. |
| *Vicia sativa* | 0.0 | 0.0 | 0.0 | 0.0 | 0.0 |

n.d.: not determined, because the species did not reach BBCH 89

-*: 100 % Mortality was already reached at the vegetative stage.
