# Supplementary material for: Feasibility of assessing vegetative and generative endpoints of crop- and non- crop terrestrial plant species for non-target terrestrial plant (NTTP) regulatory testing under greenhouse conditions
Source: PLoS One. 2020 Mar 10;15(3):e0230155. doi: 10.1371/journal.pone.0230155 (PMC7064193; doi:10.1371/journal.pone.0230155)
Supplement: S2 Text — (DOCX) [file pone.0230155.s002.docx]

**Table A. Emergence rate (in %) of the species until the application of the test substance**

| **Species** | **Emergence rate (in %)** |
| --- | --- |
| *Avena fatua* | 88.7 |
| *Brassica rapa* | 92.3 |
| *Chenopodium berlandieri* | 79.6 |
| *Coriandrum sativum* | 82.5 |
| *Fagopyrum esculentum* | 94.1 |
| *Lepidium sativum* | 89.7 |
| *Leucanthemum vulgare* | 79.7 |
| *Lolium multiflorum* | 91.4 |
| *Lotus corniculatus* | 81.7 |
| *Matricaria recutita* | 82.9 |
| *Papaver rhoeas* | 83.1 |
| *Phacelia tanacetifolia* | 89.1 |
| *Secale cereale* | 85.2 |
| *Sinapis alba* | 96.9 |
| *Trifolium pratense* | 91.8 |
| *Veronica persica* | 76.4 |
| *Vicia sativa* | 94.8 |

**Table D. Germination rate in % of harvested seeds (F1) without seed storage (sown within 14 days after the harvest).**

| **Species** | **Application rates of test substance**  **(g product ha^-1^)** | | | | |
| --- | --- | --- | --- | --- | --- |
|  | **0 (Control)** | **12** | **40** | **120** | **400** |
| *Agrostemma githago* | 0 | 0 | 0 | n.d. | n.d. |
| *Avena fatua* | 0 | 3 | 0 | 0 | 3 |
| *Coriandrum sativum* | 0 | 0 | 0 | 0 | n.d. |
| *Fagopyrum esculentum* | 93 | 87 | 87 | 90 | n.d. |
| *Lepidium sativum* | 3 | 0 | 0 | n.d. | n.d. |
| *Phacelia tanacetifolia* | 43 | 17 | 7 | n.d. | n.d. |
| *Sinapis alba* | 100 | 97 | n.d. | n.d. | n.d. |
| *Vicia sativa* | 7 | 13 | 3 | 0 | n.d. |

n.d. = value could not be determined.
